# Tumor necrosis factor superfamily member APRIL contributes to fibrotic scar formation after spinal cord injury

**DOI:** 10.1186/s12974-016-0552-4

**Published:** 2016-04-20

**Authors:** Lucy H. Funk, Amber R. Hackett, Mary Bartlett Bunge, Jae K. Lee

**Affiliations:** Department of Neurological Surgery, Miami Project to Cure Paralysis, University of Miami School of Medicine, Miami, FL 33136 USA; Department of Cell Biology, University of Miami School of Medicine, Miami, FL 33136 USA; Department of Neurology, University of Miami School of Medicine, Miami, FL 33136 USA; University of Miami School of Medicine, 1095 NW 14th Terrace, LPLC 4-19, Miami, FL 33136 USA

**Keywords:** TNFSF13, Glial scar, Cell proliferation, Fibrosis, BAFF, TACI, BCMA, BAFF-R

## Abstract

**Background:**

Fibrotic scar formation contributes to the axon growth-inhibitory environment that forms following spinal cord injury (SCI). We recently demonstrated that depletion of hematogenous macrophages led to a reduction in fibrotic scar formation and increased axon growth after SCI. These changes were associated with decreased TNFSF13 (a proliferation inducing ligand (APRIL)) expression, but the role of APRIL in fibrotic scar formation after SCI has not been directly investigated. Thus, the goal of this study was to determine the role of APRIL in fibrotic scar formation after SCI.

**Methods:**

APRIL knockout and wild-type mice received contusive SCI and were assessed for inflammatory cytokine/chemokine expression, leukocyte infiltration, fibrotic scar formation, axon growth, and cell proliferation.

**Results:**

Expression of APRIL and its receptor BCMA is increased following SCI, and genetic deletion of APRIL led to reduced fibrotic scar formation and increased axon growth. However, the fibrotic scar reduction in APRIL KO mice was not a result of changes in fibroblast or astrocyte proliferation. Rather, APRIL knockout mice displayed reduced TNFα and CCL2 expression and less macrophage and B cell infiltration at the injury site.

**Conclusions:**

Our data indicate that APRIL contributes to fibrotic scar formation after SCI by mediating the inflammatory response.

## Introduction

Developing successful therapies for spinal cord injury (SCI) is a formidable medical challenge in part because of the axonal growth-inhibitory environment that develops following injury characterized by a glial and fibrotic scar [1]. Whereas the glial scar has been an important research focus for many years, less attention has been given to the fibrotic scar. Our recent work demonstrated that hematogenous macrophages are important for the formation of the fibrotic scar [2]. Additionally, reduced fibrotic scar formation after macrophage depletion was associated with decreased expression of tumor necrosis factor superfamily member 13 (TNFSF13), also known as a proliferation inducing ligand (APRIL). Thus, our goal for this study was to determine if APRIL is necessary in the formation of the fibrotic scar following contusive SCI.

Past research on APRIL has focused mostly on its role in cancer and B cell survival and proliferation [3]. APRIL has been shown to be elevated in several diseases such as rheumatoid arthritis [4], systemic lupus erythematosus [5], multiple sclerosis [6], and schizophrenia [7]. Macrophages [8, 9], B cells, and activated T cells [10] express APRIL, and APRIL can interact with transmembrane activator and CAML interactor (TACI), B cell maturation antigen (BCMA), and heparan sulfate proteoglycan (HSPG) receptors [11–14]. These receptors are shared with another closely related tumor necrosis factor (TNF) superfamily member B cell-activating factor (BAFF, TNFSF13b) [12, 13]. Many of APRIL’s functions are attributed to activating the nuclear factor kappa-light-chain-enhancer of activated B cells (NF-κB) pathway which in turn can induce APRIL expression [15, 16]. Information on APRIL in the CNS is limited. APRIL has been shown to be expressed by hippocampal pyramidal cells and astrocytes; microglia have been shown to express TACI [17–19]. However, the role of APRIL following traumatic CNS injury has not been studied.

To test the hypothesis that APRIL is necessary for fibrotic scar formation after SCI, we performed SCI in APRIL knockout (KO) mice. We found that APRIL expression is upregulated following SCI and genetic deletion of APRIL attenuates the inflammatory response and reduces fibrotic scar area. To our knowledge, this is the first study to directly investigate the role of APRIL after traumatic CNS injury in vivo.

## Materials and methods

### Surgery and behavioral assessment

TNFSF13 KO mice in a C57BL/6 genetic background were kindly donated by Dr. Eckhard Podack. Wild-type C57BL/6 mice served as controls. Eight- to twelve-week old female mice underwent a moderate contusive SCI as previously described [20]. Briefly, anesthetized mice (ketamine/xylazine, 100 mg/15 mg/kg i.p.) received a T8 laminectomy followed by a moderate contusive SCI (65 kDynes) using an Infinite Horizon Impactor (Precision Systems and Instrumentation, LLC). Post-operative treatment for the first week included twice daily injections of Lactated Ringer’s solution (1 ml), an antibiotic (Baytril, 10 mg/kg), and an analgesic (buprenorphine, 0.05 mg/kg) subcutaneously. Twice daily bladder expressions continued for the duration of the study. Locomotor recovery was assessed using the Basso Mouse Scale [21] open-field test at 1 day and weekly after injury. Only mice with a BMS score of 0 or 1 at 1 day after injury were included in the study. To identify proliferating cells, 50 mg/kg 5-ethynyl-2′-deoxyuridine (EdU) (Invitrogen A10044) was injected intraperitoneally on days 3, 4, and 5 following SCI. All procedures were in accordance with University of Miami Institutional Animal Care and Use Committee (IACUC) and National Institutes of Health (NIH) guidelines.

### Gene expression analysis

Mice were anesthetized as described above and perfused transcardially with cold Dulbecco’s phosphate-buffered saline (DPBS, Gibco 14190-144). Naïve or wild-type (WT) injured mice were used as controls. A 4-mm section of the spinal cord injury site, or the corresponding T8 spinal cord in naïve controls, was dissected and then homogenized for RNA extraction using the Qiagen RNeasy Plus Micro kit (74034). Subsequently, cDNA was synthesized using the Advantage RT-for-polymerase chain reaction (PCR) kit (Clontech 639506). cDNA (0.5 μg per sample) was added to RT^2^ SYBR Green Mastermix and PCR performed using Eppendorf Mastercycler ep realplex. Primer sequences were as follows (5′-3′): APRIL (forward AATTCTCCTGAGGCTAGGGGG; reverse AGGACATCAGGACTCTGCTCC), BAFF (forward CGACACGCCGACTATACGAA; reverse GGTCCGTGTATAGAACCTGGC), BCMA (forward ACTTGCGATGTTCCAACCCT; reverse ATCCGTCAAGCTGACCTGG), TACI (forward ACGTGTAGCTTCTGCTTCCC; reverse CGCCTACTGTTGCACGCATA), BAFF-R (forward CCAGTGCAATCAGACCGAGT; reverse GGGTTTCTGAGGAGGGTACAA), GAPDH (forward TGGCCTTCCGTGTTCCTAC; reverse GAGTTGCTGTTGAAGTCG), TNF (forward AGGCACTCCCCCAAAAGATG; reverse TCACCCCGAAGTTCAGTAGAC), CCL2 (forward CCCCACTCACCTGCTGCTAC; reverse CCTGCTGCTGGTGATTCTCTT), IL-1β (forward CTTCAAATCTCACAGCAGCACATC; reverse CCACGGGAAAGACACAGGTAG), CXCL10 (forward GCCGTCATTTTCTGCCTCATCCT; reverse CTCATTCTCACTGGCCCGTCATC), CCL5 (forward TGCCCACGTCAAGGAGTATTTCTA; reverse TGGCGGTTCCTTCGAGTGACAA), and IL-6 (forward AACCACGGCCTTCCCTACTTCA; reverse TCATTTCCACGATTTCCCAGAG ).

### Histology

Mice were anesthetized and perfused transcardially with 4 % paraformaldehyde 2 weeks post-SCI. Spinal cords were dissected and post-fixed for 2 h before placing in 30 % sucrose overnight. An 8-mm spinal segment centered at the injury site was embedded in OCT compound (Tissue-Tek) and sectioned sagittally on a cryostat into 16-μm serial sections. Sections were subsequently immunostained with primary antibodies for glial fibrillary acidic protein (GFAP) (Invitrogen 130300, 1:500), Neurofilament medium (EnCor Biotechnology, RPCA-NF-M, 1:500) and platelet-derived growth factor receptor beta (PDGFRβ) (Abcam plc, ab32570, 1:200) in PBS-0.3 % Triton X-100. The sections were then incubated with the corresponding Alexa Fluor secondary antibodies (Invitrogen, 1:500). Sections were mounted in VECTASHIELD containing DAPI (Vector Laboratories), and images were collected with a Nikon Eclipse Ti fluorescent microscope. EdU labeling was visualized using Life Technologies Click-iT EdU imaging kit (Life Technologies, C10340).

### Quantification

Quantification of immunohistochemical images was performed using ImageJ by observers blinded to the experimental groups. The fibrotic scar size was measured by tracing the PDGFRβ^+^ area within a 2-mm segment of the spinal cord centered on the injury site. The PDGFRβ^+^ area was expressed as a percentage of the area of the 2-mm spinal cord segment evaluated. To quantify the number of neurofilament^+^ axons and EdU^+^ fibroblasts, 50-μm square grids were generated over the injury site and every sixth square in the GFAP^−^ region was quantified. To quantify the EdU^+^ astrocytes, every third square in the grid within the 250-μm border region of the glial scar was evaluated. For each animal, sections including the injury epicenter and two adjacent sagittal sections spaced 128 μm apart were quantified, and the counts from each section were averaged.

### Flow cytometry

A cell suspension of the spinal cord 1 week following injury underwent myelin removal (myelin removal beads, Miltenyi Biotec). Subsequently, cells were resuspended in 100-μl FACS buffer and Fc blocked with anti-mouse CD16/32 (BioLegend, 1:200) for 10 min on ice and were then incubated for 30 min at 4 °C with anti-CD45-APC/Cy7, anti-CD11b-APC, anti-B220-FITC, and anti-CD3-PE/Cy7. Cell suspensions were analyzed as previously described [2]. Cell numbers were quantified using 123count ebeads (eBioscience).

## Results

**Fig. 1:**
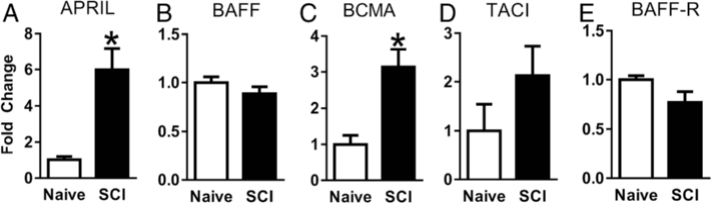
mRNA expression of APRIL, BAFF, and their receptors at 2 weeks after contusive spinal cord injury. In the injured spinal cord, APRIL expression is significantly increased whereas its related ligand BAFF is unaltered **(a–b)**. Of the APRIL family receptors, BCMA **(c)** expression is significantly increased but TACI **(d)** and BAFF-R **(e)** are not changed. *n* = 5 per group (biological replicates). Normalized to average of naïve for each gene. *p < 0.01 compared to naïve. Student’s *t* test

**Fig. 2:**
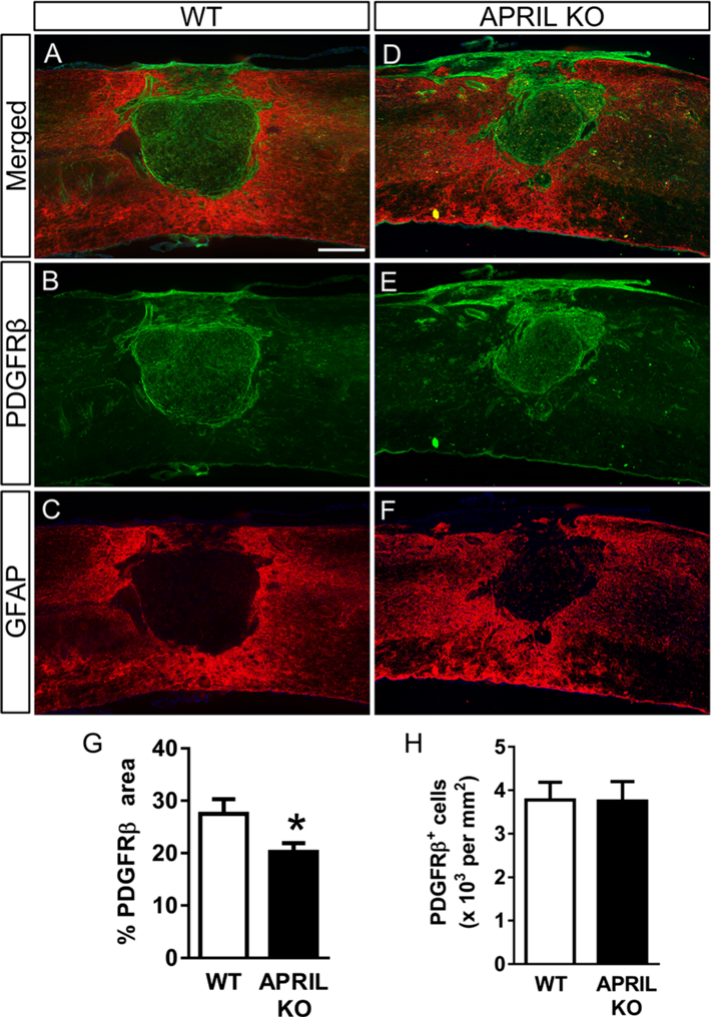
APRIL KO mice have a reduced fibrotic scar area after SCI. Two weeks following SCI, the fibrotic scar size was assessed in APRIL KO (**d**–**f**, *n* = 9) and WT (**a**–**c**, *n* = 11) mice by evaluating the percent area of PDGFRβ signal within a 2-mm region centered around the injury site **(g)**. The density of PDGFRβ^+^ cells was not different between WT and KO mice **(h)**. Representative images from a WT and APRIL KO animal are shown with GFAP in *red* and PDGFRβ in *green. n* = biological replicates; *p < 0.05 compared to WT. Student’s *t* test. Scale bar = 250 μm

**Fig. 3:**
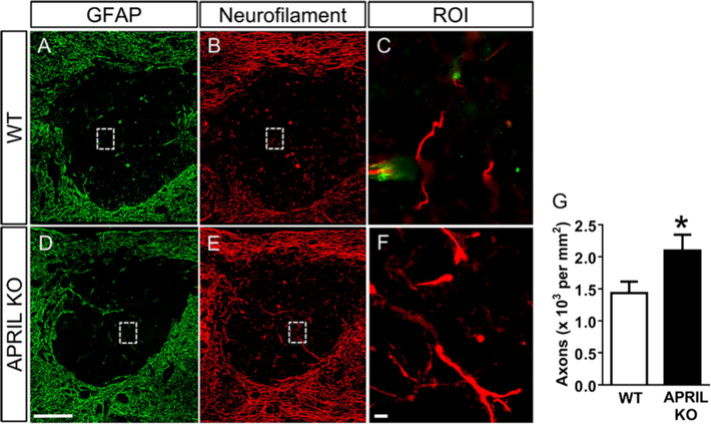
APRIL KO mice have an increased number of axons in the injury site 2 weeks following SCI. Neurofilament^+^ axons (*red*) in the GFAP (*green*)-negative region were compared between WT **(a–c)** and APRIL KO **(d–f)** mice 2 weeks following injury. **c**, **f** Magnified images of *dotted area* (region of interest (ROI)). Quantifications are shown in **g** (*n* = 8 biological replicates). Scale bar = 250 μm **(a, b, d, e)**, 10 μm **(c, f)**. *p < 0.05 compared to WT. Student’s *t* test

The expression of APRIL, BAFF, and their receptors in the spinal cord was evaluated using qRT-PCR following SCI. Expression of APRIL was increased at 14 days after SCI (Fig. 1a). The APRIL receptor BCMA, but not TACI, was also significantly increased following SCI (Fig. 1c, d). However, expression of the related ligand BAFF and BAFF-R was not altered following SCI (Fig. 1b, e). To evaluate the role of APRIL in the formation of the fibrotic scar, we measured PDGFRβ^+^ areas at 2 weeks after SCI. We previously demonstrated that PDGFRβ is a reliable marker of fibroblasts at the SCI site [20]. We chose the 2-week time point because this is when the scar starts to mature and take its final shape. In both WT and APRIL KO mice, PDGFRβ^+^ fibroblasts were present throughout the GFAP^−^ region of the injury site with a dense boundary apposing the astroglial scar (Fig. 2a–f). However, at 2 weeks after SCI, the PDGFRβ^+^ area in APRIL KO mice was significantly reduced (Fig. 2d–g), whereas the fibroblast density remained the same (Fig. 2h), suggesting that APRIL contributes to fibrotic scar formation after SCI. To determine the effect of this reduced fibrotic scar on axon growth, we measured the number of neurofilament-positive axons in the fibrotic scar region. APRIL KO mice had more axons in the fibrotic scar compared to wild-type mice, suggesting that genetic deletion of APRIL promotes axon growth by reducing the fibrotic scar (Fig. 3).

**Fig. 4:**
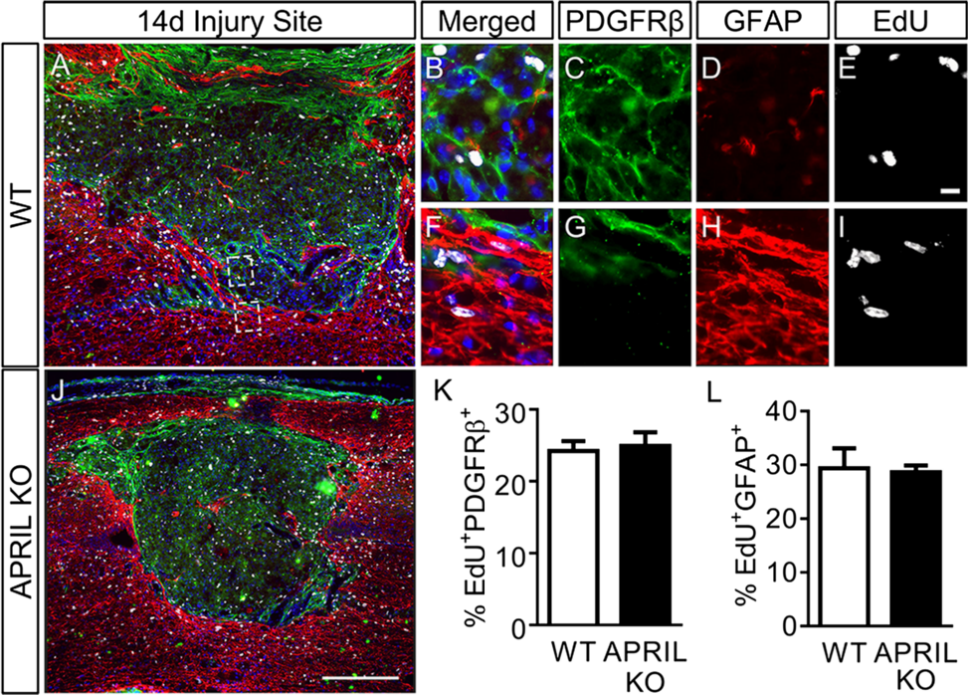
Proliferation of fibroblasts and astrocytes is not altered in APRIL KO mice after SCI. EdU^+^ cells (*white*) are widely distributed throughout the GFAP (*red*) and PDGFRβ (*green*) regions in APRIL KO **(j)** and WT **(a–i)** animals. High-magnification images of proliferating fibroblasts **(b–e)** and astrocytes **(f–i)** are shown. Quantification of proliferating fibroblasts **(k)** and astrocytes **(l)** reveals no difference between APRIL KO and WT mice following SCI. **b**–**e** From *top dashed square region* in **(a). f**–**i** From *bottom dashed region* in **(a)**. Percent in **k** and **l** are calculated from the total number of PDGFRβ^+^ or GFAP^+^ cells within the quantified region. *n* = 3 per group (biological replicates). Scale bar = 250 μm **(a, j)**, 10 μm **(b–i)**

To determine if the reduced fibrotic scar size was due to decreased fibroblast proliferation, EdU was injected on days 3, 4, and 5 following injury. These time points have been previously shown to be the peak of glial cell proliferation as well as when fibroblasts start to appear after SCI [20, 22]. Two weeks after SCI, there was no difference between WT (Fig. 4a–i) and APRIL KO (Fig. 4j) animals in the percentage of EdU^+^ fibroblasts (Fig. 4k), demonstrating that the smaller fibrotic scar was not due to a reduction in fibroblast proliferation in APRIL KO mice. To determine if the smaller fibrotic scar could be due to an increased glial scar size resulting from increased astrocyte proliferation, the number of EdU^+^GFAP^+^ cells was counted in the 250-μm border of the glial scar (Fig. 4). The number of EdU^+^ astrocytes in the glial scar border was not different between WT and APRIL KO mice (Fig. 4l), suggesting that astrocyte proliferation was not significantly affected after genetic deletion of APRIL after SCI. Taken together, our data indicate that the reduced fibrotic scar size is not due to changes in fibroblast or astrocyte proliferation after SCI.

**Fig. 5:**
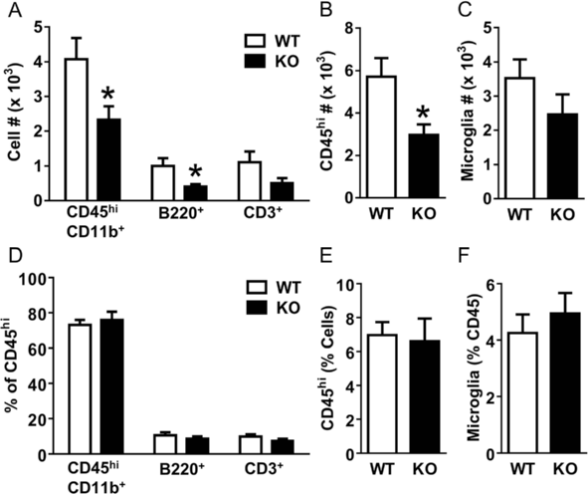
The number of infiltrating leukocytes is decreased in APRIL KO mice 1 week following SCI. The number of infiltrating macrophages (CD45^hi^CD11b^+^) and B cells (B220^+^) were significantly reduced following SCI in APRIL KO mice (*n* = 7) compared to WT controls (*n* = 10) **(a)**. This translated to a reduction in the overall number of infiltrating leukocytes **(b)**, whereas the number of microglia remained similar between WT and KO mice **(c)**. However, the percentages of each cell type, including microglia, were not different **(d–f)**. *n* = biological replicates. *p < 0.05 compared to WT. Student’s *t* test

We had previously demonstrated that macrophages are an important mediator of fibrotic scar formation after SCI [2]. Thus, to consider the possibility that the reduced fibrotic scar in APRIL KO mice was an indirect result of the effect of APRIL on macrophage recruitment, we assessed leukocyte infiltration in APRIL KO mice. At 7 days after SCI, we dissociated the injury site tissue from APRIL KO and wild-type mice and performed flow cytometry to assess the number of macrophages (CD45^hi^CD11b^+^), microglia (CD45^low^CD11b^+^), B cells (B220^+^), and T cells (CD3^+^). We chose the 7-day time point because this is the peak of macrophage infiltration [23]. Whereas the percentages of each cell type were similar between wild-type and APRIL KO mice (Fig. 5d–f), the number of macrophages and B cells was significantly reduced (Fig 5a–c). This translated into a reduction in the overall leukocyte number (CD45^hi^) at the injury site (Fig. 5b). However, the number of microglia and T cells was not different between the two groups. Therefore, the reduced fibrotic scar in APRIL KO mice was associated with reduced macrophage and B cell infiltration after SCI.

**Fig. 6:**
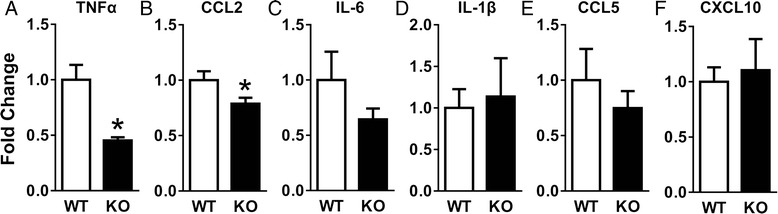
Cytokine/chemokine expression is decreased in APRIL KO mice 1 day following SCI. Injury sites from APRIL KO mice had significantly decreased expression of TNFα **(a)** and CCL2 **(b)** compared to wild-type controls. IL6, IL-1β, CCL5, and CXCL10 expression was unchanged **(c–f)**. *n* = 7–8 biological replicates per group. Normalized to average of WT for each gene. *p < 0.05 compared to WT. Student’s *t* test

To determine whether the reduced leukocyte infiltration could be due to altered cytokine/chemokine expression acutely after injury in APRIL KO mice, we used qRT-PCR to assess the expression of multiple inflammatory cytokines/chemokines at 1 day after SCI, which is considered the peak of inflammatory cytokine/chemokine expression [24]. We found that APRIL KO mice have reduced expression of TNFα and CCL2 compared to wild-type controls (Fig. 6a, b), indicating that APRIL contributes to the acute expression of inflammatory cytokines/chemokines after SCI and raising the possibility that this reduced acute inflammatory response may have eventually led to reduced leukocyte infiltration that resulted in attenuated fibrotic scar formation.

## Discussion

Our goal in this study was to test the role of APRIL in fibrotic scar formation following SCI. We demonstrated that APRIL and BCMA expression is increased following SCI and that genetic deletion of APRIL leads to a reduced fibrotic scar area that is associated with increased axonal growth. This reduction, however, was not due to altered proliferation of fibroblasts or astrocytes at the injury site, which was surprising given the known role of APRIL in cancer metastasis and reports of APRIL affecting proliferation of fibroblast-like synoviocytes and astrocytes [18, 25]. In addition, the density of fibroblasts was not different between WT and APRIL KO mice, suggesting that reduced fibrotic areas was not due to compaction of this region, but rather to a reduced overall number of fibroblasts recruited to the injury site.

Thus, we pursued the possibility that rather than acting directly on fibroblasts, APRIL could be mediating fibrotic scar formation indirectly through macrophages. Indeed, we found significantly reduced macrophage number at the injury site of APRIL KO mice. In addition, we also found a reduced number of B cells, which was expected given the well-known role of APRIL in regulating B cell proliferation. This reduction in leukocyte infiltration was associated with reduced TNFα and CCL2 expression in APRIL KO mice, suggesting that the reduced leukocyte infiltration was due, at least in part, to the regulation of the acute inflammatory response by APRIL. Taken together, our data suggest that APRIL regulates the acute expression of pro-inflammatory cytokines that leads to recruitment of leukocytes to form the fibrotic scar after SCI. However, we cannot rule out the possibility that these processes occur in parallel with direct effects of APRIL on leukocyte infiltration and/or fibrotic scar formation.

Our finding that TNFα and CCL2 expression is decreased 1 day after SCI in APRIL KO mice suggests that APRIL is a regulator of the acute inflammatory response. Since macrophages and lymphocytes are not a major cellular component of the injury site at this early time point, the most likely sources of APRIL are neutrophils, microglia, and/or astrocytes. The mechanism by which APRIL is expressed by these cells regulates cytokine expression after SCI remains to be determined. In conclusion, our data indicate that APRIL contributes to fibrotic scar formation after SCI by mediating the acute inflammatory response and recruitment of leukocytes.

### Ethics approval and consent to participate

All animal procedures were in accordance with University of Miami IACUC and NIH guidelines. There are no human participants, data, or tissue in this study.

### Availability of data and materials

All datasets on which the conclusions of the manuscript rely are presented in this manuscript.

## Abbreviations

APRIL: a proliferation inducing ligand
BAFF: B cell-activating factor
BAFF-R: B cell-activating factor receptor
BCMA: B cell maturation antigen
BMS: Basso Mouse Scale
EdU: 5-ethynyl-2′-deoxyuridine
HSPG: heparan sulfate proteoglycan
KO: knockout
NF-κB: nuclear factor kappa-light-chain-enhancer of activated B cells
PCR: polymerase chain reaction
PDGFRβ: platelet-derived growth factor receptor beta
ROI: region of interest
SCI: spinal cord injury
TACI: transmembrane activator and CAML interactor
TNF: tumor necrosis factor
TNFSF: tumor necrosis factor superfamily
WT: wild-type
